# Sonar beam dynamics in leaf-nosed bats

**DOI:** 10.1038/srep29222

**Published:** 2016-07-07

**Authors:** Meike Linnenschmidt, Lutz Wiegrebe

**Affiliations:** 1 Ludwig-Maximilians-University Munich, Division of Neurobiology, Dept. Biology II, Großhaderner Str. 2, 82152 Planegg-Martinsried, Germany

## Abstract

Ultrasonic emissions of bats are directional and delimit the echo-acoustic space. Directionality is quantified by the aperture of the sonar beam. Recent work has shown that bats often widen their sonar beam when approaching movable prey or sharpen their sonar beam when navigating through cluttered habitats. Here we report how nose-emitting bats, *Phyllostomus discolor*, adjust their sonar beam to object distance. First, we show that the height and width of the bats sonar beam, as imprinted on a parabolic 45 channel microphone array, varies even within each animal and this variation is unrelated to changes in call level or spectral content. Second, we show that these animals are able to systematically decrease height and width of their sonar beam while focusing on the approaching object. Thus it appears that sonar beam sharpening is a further, facultative means of reducing search volume, likely to be employed by stationary animals when the object position is close and unambiguous. As only half of our individuals sharpened their beam onto the approaching object we suggest that this strategy is facultative, under voluntary control, and that beam formation is likely mediated by muscular control of the acoustic aperture of the bats’ nose leaf.

The environment for bats and toothed whales has limited natural illumination, thus echolocation^1^ becomes their most essential sensory modality for foraging and orientation, often superior to vision. In echolocation, the distance to an object is explicitly encoded in the delay of the object’s echo relative to the sonar emission. Echo delay decreases linearly with decreasing object distance. While bats adjust both inter-call interval and call duration^1^,^2^,^3^,^4^,^5^ to object distance, most toothed whales adjust only the click interval^6^,^7^. Similar to bats, they thus avoid a temporal overlap between the returning echo and the next emission. Only beaked whales have shown a reduction in call duration when changing from FM clicks to buzz clicks^8^. Moreover, all bats and toothed whales studied to date reduce the sound pressure level of their emissions with decreasing object distance^2^,^9^,^10^,^11^,^12^,^13^, likely in an attempt to keep the echo level in a physiological range.

Sonar emissions are directional, i.e., the emission level depends on the azimuth and elevation from which it is measured. This spatial projection pattern, generally referred to as the sonar beam, has been quantified both for bats^14^,^15^,^16^ and toothed whales^7^,^17^,^18^,^19^. A sharper beam improves the acoustic focus onto an object because the ensonified space ahead is narrowed which reduces reflections from obstacles to the side and object localisation improves. At the same time detection range increases if the emission level is kept constant as a higher proportion of sound energy is directed along the acoustic axis^20^. Foraging vespertilionid and rhinolophid bats utilise this relationship in that they broaden the sonar beam shortly before catching insects in flight^13^,^21^,^22^. This reduces the detection range but increases the ensonified airspace, which at this point probably allows the bats to keep better track of the fast-moving and evasive prey. In vespertilionids, beam broadening is achieved by the bats lowering the spectral centroid of their emission, which, at a fixed aperture of the sound-emitting structure, broadens the beam^14^,^23^,^24^. Recent work shows that mouth emitting bats (*Hypsugo bodenheimeri*) can also change mouth gape to define their sonar beam directionality according to the amount of clutter echoes they encompass^25^. The nose-emitting rhinolophid bats do not change the spectral centroid of their signals during prey capture. Instead, the authors postulate that rhinolophids broaden their beam by adjustments of the nose leaf surrounding the nostrils^22^,^26^. Similar muscular control of the sound projecting structures and the subsequent sonar-beam broadening appears in harbour porpoises during the final stage of capturing fish^19^. The melon, a fatty tissue in the forehead of toothed whales, is surrounded by muscles^27^, which are probably able to adjust the shape of the melon that transmits the echolocation signal into the water^19^. In that study, harbour porpoises broadened their sonar beam during capture of fish, likely for the same reasons as beam-broadening in bats, i.e., to keep the fast-moving prey within the ensonified space.

Phyllostomid bats, like rhinolophid bats, emit their signals through a pair of nostrils surrounded by a conspicuous nose leaf, which often extends at the top in the shape of a lancet. It has been demonstrated that phyllostomid bats in general produce highly directional sonar beams^15^,^28^. Earlier studies with restrained phyllostomid bats in which the nose leaf was manipulated and modelling studies have shown that the nose leaf determines especially the vertical directionality^29^,^30^ while the nostril spacing determines horizontal directionality^29^ Furthermore, it has been suggested Macrophyllum macrophyllum can bend its nose leaf intentionally^31^. This would certainly affect the beam directionality. Thus it has been speculated several times that phyllostomid bats may be able to change the sonar beam shape by means of the nose leaf. However, no evidence for sonar beam dynamics in phyllostomid bats has been formally presented.

Here we quantify the extent to which nose-emitting bats (*Phyllostomus discolor*) show individual sonar beam dynamics and how they utilise such dynamics while ensonifying an approaching object.

## Material and Methods

### Animals and experimental paradigm

**Figure 1:**
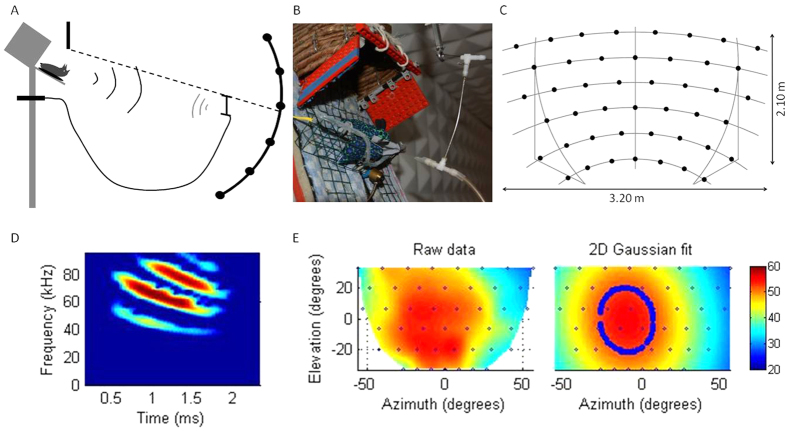
Overview of set up and analysis illustration. (**A**) Simplified diagram of the experimental set up. The bat on the stationary platform (left) echo-acoustically tracked the approaching object with the microphone array to the right. (**B**) Positioning of bat (dummy bat) on platform between house and real target (T-connector). (**C**) Microphone array with microphone positions (black dots). (**D**) Sonogram of *P. discolor* echolocation call. (**E**) Footprint of sonar beam before (left) and after fitting a 2D Gaussian function (right). The circle given by the blue markers defines the −3 dB sonar beam area (in this example are beam height 40°, beam width 34° and the goodness of fit R^2^ 0.95). Call level is colour coded and spans a range of 40 dB from dark blue to red.

Six adult male *P. dicolor* bats were trained to sit on a platform (PVC 16 × 22 × 2 cm) at a height of 1.8 m and an angle of 70° (Fig. 1A). The bats were trained to echolocate towards an approaching food source (PVC T tube-connector, 3 × 0.5 cm, attached to a metal stick, 10 × 0.1 cm; Fig. 1B). A flexible PVC tube (200 × 0.4 cm) was attached at the rear of the T-connector and to a motor driven syringe (20 ml) mounted below the platform. A nylon string (0.05 mm diameter) was stretched between the centre of the microphone array and the platform. The object was moved along this nylon string from >1.0 m distance towards the bat. A small enclosure (15 × 15 × 15 cm) made of banana leaves at the end of the platform provided shelter for the bat. A small motorized door with two rotating door wings (each 15 × 7 cm; Lego Technic, LEGO A/S, Billund, Denmark Fig. 1B) was placed within the opening of the shelter. The experiment took place in a completely dark, echo-attenuated chamber (2.2 × 2.2 × 3.6 m). Experimental procedure and data collection were controlled through a graphical user interface (Matlab v.7.5, MathWorks, Cambridge, MA, USA) on a desktop computer located outside the chamber. Two infrared sensitive surveillance cameras served to monitor the bats on the platform and the positioning of the object.

We ran one or two sessions per day for five days of the week. A session lasted between 10 and 30 min and consisted of 10 to 30 trials, depending on the motivation of the animals. The beginning of a trial was signalled to the bat by opening the doors. The object was moved towards the bats within a time window of 2 s once the bats stepped outside the shelter and started to echo-acoustically search for the object. The object distance was manually controlled by means of a nylon string attached to the object. A drop of banana pulp was provided by the object upon arrival at the bat. To start a new trial, the bats had to move back into the house and the doors were closed again.

This experiment was conducted under the principles of laboratory animal care and the regulations of the German Law on Animal Protection. The license to keep and breed *P. discolor* as well as all experimental protocols were approved by the German Regierung von Oberbayern.

### Acoustic recordings and analysis

Echolocation calls were recorded with a 45 channel parabolic microphone array (SPM0204uD5, Knowles Corporation, Itasca, IL, USA) at 1.9 m distance (Fig. 1C). The array covered 65° elevation and 112° azimuth. The microphone carrier construction consists of six horizontal and three vertical parabolic aluminum bars (2.0 × 2.0 cm; Item, Industrietechnik GmbH, Solingen, Germany). To each of the horizontal bars were either 8 or 7 microphones mounted with inter-microphone distances of 53, 54, 51, 46, 40, 31, and 43 cm for the horizontal bars 1–6, respectively. Each microphone channel was separately amplified (55 dB; Octopre MKII, Focusrite, High Wycombe, England), digitized (sampling rate = 192 kHz, 24-bit resolution; HD192, Motu, Cambridge, MA, USA) and recorded into a 2 s ring-buffer. Matlab based sound recording was programmed with the SoundmexPro Toolbox (Hoertech, Oldenburg, Germany). The sigma-delta converters of the HD192 do not require extra anti-aliasing filters. A separate analogue to digital board (USB1208LS, Measurement Computing, Norton, MA, USA) sampled at 50 Hz the voltage proportional to the object distance. This voltage was generated via a 10 gauge potentiometer that was rotated by the string that controlled object distance. Data acquisition was stopped automatically once the object was at the bat’s position and the reward was given. The microphone recordings were stored together with the digitized distance readings in a .mat file.

Analysis was done with custom written programs (Matlab v. 2010b, MathWorks, Cambridge, MA, USA). For each trial the 45 channel recording was compensated with a finite impulse response (supplementary information, supplementary methods), referenced against a Brüel & Kjær 4135 ¼ inch microphone (Brüel & Kjær, Nærum, Denmark). Then recordings were high pass filtered at 35 kHz and extracted echolocation calls in each channel were aligned with the object distance. Call level, temporal call parameters (inter-call interval and −10 dB call duration), and spectral call analysis (spectral centroid) were done from the microphone with the highest call level. Call level was calculated for each call centered within a 2.5 ms rectangular window. The pattern of sound levels of a call across the microphone array was fitted with a 2D Gaussian function (Fig. 1E). From the fit, the −3 dB sonar beam height and width were extracted. The following data-acceptance criteria were applied: 1. Initial object distance at the start of a trial was at least 1 m, 2. Echolocation calls had to have a signal to noise ratio of >10 dB at the microphone with the highest call level, 3. The entire −3 dB sonar beam had to be on the array, 4. The goodness of fit value for the 2D Gaussian fit, quantified as the percentage of explained variation, ‘R squared’, had to be >0.8. If single echolocation calls did not fulfil theses 4 criteria, the calls were excluded from further analysis.

**Figure 2:**
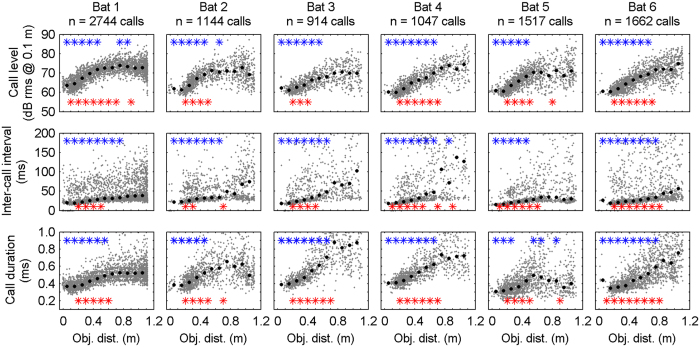
Amplitude- and temporal properties of echolocation calls. Distribution of call level (row 1), inter-call interval (row 2) and call duration (row3) as a function of object distance. Data are given by single call values (grey dots) and medians of pooled data in 10 cm bins (black diamonds). Significant differences (p ≤ 0.05, Wilcoxon rank test) between (bin (x)) relative to the reference bin at 95 cm are represented by the upper (blue) stars and between neighbouring bins (bin (x) and bin (x + 1)) are given by the lower (red) stars.

**Figure 3:**
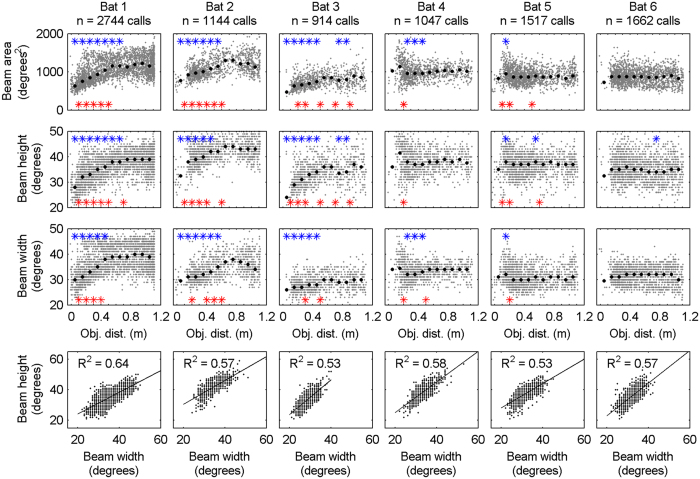
Spatial analysis of sonar beams. Distribution of −3 dB beam area (row 1), −3 dB beam height (row 2) and −3 dB beam width (row 3) as a function of object distance. Data are given by single call values (grey dots) and medians of pooled data in 10 cm bins (black diamonds). Significant differences (p ≤ 0.05, Wilcoxon rank test) between bin (x) relative to the reference bin at 95 cm are represented by the upper (blue) stars and between neighbouring bins (bin (x) and bin (x + 1)) are given by the lower (red) stars. Note that there is a significant correlation between beam height and width for all six bats (lower row, p ≤ 0.001).

**Figure 4:**
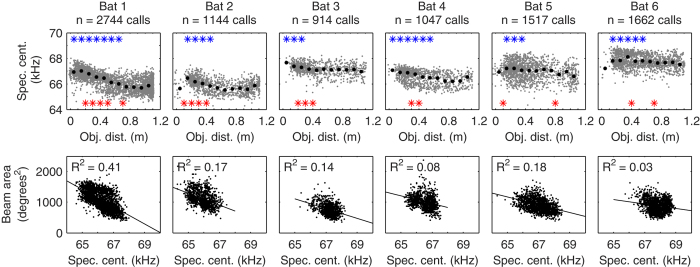
Spectral analysis of echolocation calls. Distribution of call spectral centroid as a function of object distance (upper row). Data are given by single call values (grey dots) and medians of pooled data in 10 cm bins (black diamonds). Significant differences (p ≤ 0.05, Wilcoxon rank test) between bin (x) relative to the reference bin at 95 cm are represented by the upper (blue) stars and between neighbouring bins (bin (x) and bin (x + 1)) are given by the lower (red) stars. Note that while the correlation between beam area and call spectral centroid are significant (lower row, p ≤ 0.001), the correlation coefficient (R^2^) is quite small, indicating only a small change of spectral centroid with a large change in beam area.

Data were pooled into 10 cm bins centered at 5, 15, … and 105 cm. Significant differences (p ≤ 0.05, Wilcoxon rank sum test) between bin (x) relative to the reference bin at 95 cm are represented in Figs 2, 3, 4 by the upper (blue) stars and between neighbouring bins (bin(x) and bin (x + 1)) are given by the lower (red) stars.

## Results

Six bats performed between 67 and 105 trials with an object starting distance of more than 1 m each and between 43 to 66% of their echolocation calls (914 to 2744 calls per animal) passed the inclusion criteria. While the object was pulled towards the platform, the bats tracked the object’s position in a continuous manner using echolocation. Each bat significantly reduced the call level, inter-call interval, and call duration with decreasing object distance (Fig. 2). Significant differences relative to the reference distance at 95 cm are indicated by the blue stars and between neighbouring bins by the red stars (p ≤ 0.05, Wilcoxon rank sum test). When object distance decreased from >1 m to 0 m, the median reduction in call level was 11.1 dB rms @0.1 m (range 9.3–13.8 dB; Fig. 2, row 1). Rows 2 and 3 of Fig. 2 show that the bats also decreased the median inter-call interval by 36.8 ms (range 17.9–120.0 ms) and the median call duration by 0.3 ms (range 0.2–0.5 ms) while the ensonified object approached. Such adaptations in the temporal parameters of the echolocation activity with respect to object distance are known to avoid a temporal overlap between the returning echo and the following call emission. Although all bats reduced inter-call intervals in a consecutive order, the lowest median value was just below 20 ms at the shortest distances. Hence, the call repetition rates never dropped to such short intervals as described for a final buzz II during prey capture for bats hunting flying insects^32^,^33^.

The sonar beam height and width in this study were on average 36.7° (STD +/− 5.17) and 33.3° (STD +/−5.15), respectively, however, both sonar beam parameter vary strongly even within each animal. All bats showed a very wide range with the maximum beam heights and widths ranging between 21 and 59 degrees (Fig. 3, row 2 to 4). This implies that the sonar beam in *P. discolor* is highly flexible. Moreover, beam height and width remain well correlated over the large range of sonar beam dimensions (R^2^ = 0.53–0.64, p ≤ 0.001; Fig. 3, bottom row).

Figure 3 (top row) shows the angular area of the sonar beam as a function of object distance. Three bats sharpened their sonar beam with decreasing object distance while for the remaining three animals sonar beam shape was unaffected by distance. For those animals that employ sonar beam sharpening, it is statistically significant, relative to the 95 cm reference distance (blue stars in the figure) and between neighbouring bins (red stars in the figure). Beam sharpening is seen both in beam height and width (Fig. 3, rows 2 and 3). Here, the median beam height changes of 12 degree (39–28, 44–32, and 37–24 for bat 1 to 3 respectively) were slightly greater than the median beam width changes of 8 degree (43–30, 38–29, 30–26, and 36–32 for bat 1 to 4 respectively). The results of an experiment with a loudspeaker at the bats’ position broadcasting random noise confirm that none of the effects described in this study were affected by the experimental setup. Neither the speaker level nor the projection pattern of the loudspeaker was affected by the approaching object (supplementary information, supplementary methods and Fig. SI1).

Changes of beam area in FM bats have been attributed to either changes in the frequency composition of the calls emitted through a constant emission aperture^21^, or to changes of the aperture by e.g. changing mouth gape^25^. To assess the strategy used by our nose-emitting bats, we analysed the spectral centroid, which is on average 66.8 kHz (STD ± 0.86). Call spectral centroid as a function of object distance is plotted in Fig. 4 (top row). Data show that for all bats, we see a significant increase in the spectral centroid with decreasing object distance (indicated by the blue stars in Fig. 4 upper row). This increase is qualitatively consistent with a frequency-based beam sharpening. Also we observe a significant correlation between beam area and spectral centroid (p ≤ 0.001; Fig. 4, lower row). However, the median change in spectral centroid was only 0.7 kHz (0.2–1.3 kHz). Such a small variation cannot account for the beam changes observed in this study (see discussion for further details). This is supported by the fact that although all six bats showed a similar increase in spectral centroid with decreasing distance, only half of the bats demonstrated a simultaneous sharpening of the sonar beam. Thus, the results show that the small changes in the spectral content play only a minor role in shaping the sonar beam in our bats.

## Discussion and Conclusions

The current experiments corroborate previous findings for leaf-nosed bats^12^,^15^,^31^ in that all six bats significantly reduce the sound level, inter-call intervals and call duration with decreasing object distance (Fig. 2). These results are in line with the current understanding of signal design adaptations to changing distances of interest, both for other bats and toothed whales^34^,^35^. Adjustments of sound level and call duration as well as the right timing of sonar emissions optimise neural analysis of echo delay.

The parabolic microphone array allowed us to compute high-resolution footprints of the sonar beam. The fixed distance of 1.9 m between animal and each microphone simplified the analysis and reduced possible analytical errors for distance compensation. The spectral emphasis on the higher (3^rd^ and 4^th^) harmonics (Fig. 1D) in the echolocation signal of *P. discolor* predicts a narrow, and thus highly directional, sonar beam because higher frequencies project in a narrower beam than do low frequencies^36^. This was confirmed by the average 37° sonar beam height and 33° sonar beam width (−3 dB double sided amplitude drop).

Most studies describing the sonar beam of bats are given at a −6 dB single sided amplitude drop (see, however, Ghose and Moss^37^ and Kounitsky *et al*.^25^). In toothed whales, sonar-beam studies typically report the double-sided −3 dB beam width and height^19^,^38^. The elevation covered by our array (65°), together with our strict data-acceptance criterion that the entire sonar beam had to be on the array, limited the analysis and thus we chose to analyse the −3 dB double-sided beam width and height. In order to compare our data with the literature we have calculated for that animal where we have the most data (Bat 1), both the double-sided −3 dB and the single-sided −6 dB amplitude drop. Although we lost 80% of the data when switching from −3 dB to −6 dB, we were able to compare the two measures and thus estimate a conversion factor of about 0.73, i.e., the single-sided −6 dB measure was about 0.73 times the double-sided −3 dB measure. Applying this conversion factor to our average sonar beam measures we get an approximate −6 dB single-sided beam height of 27° and beam width of 24°. These results corroborate earlier laboratory studies, which demonstrated that phyllostomid bats generate a more directional sonar beam than vespertilionid bats^15^,^20^,^28^. This is also reflected by the calculated directivity index of 14 dB for *P. discolor* based on the formula given by Jacobsen *et al*.^20^, a speed of sound at 20 °C of 342 m/s, an average spectral centroid frequency of 66.8 kHz, and an assumed piston radius of 4 mm^30^. Keeping in mind that this is an approximation based on a single sound source model, the calculated directivity index of 14 dB fit well into the directivity index overview given by Jakobsen *et al*.^20^, Fig. 3, which shows a higher directivity index for phyllostomid bats than for vespertillionid bats. The directivity index compares the bat’s sound intensity along the acoustic main axis to that of an omni- directional sound source of equal acoustic power.

Although we report average sonar beam measures across all individuals it is important to note that the general sonar beam can vary among individuals. At this point we can only speculate that this might be, among other reasons, due to an intra species individual variation in transmitter size (nostril spacing and size of surrounding nose leaf) and the preferred emphasis on the 3^rd^ or 4^th^ harmonic of the individuals’ echolocation calls. Independent of the variation among individuals, all six animals demonstrate a large variation in beam height and width which shows that the sonar beam of *P. discolor* is a highly dynamical system. A flexible sonar beam is advantageous because it enables the bats to control the volume of ensonified airspace. Thus it allows the bats to optimally adjust their sonar beam to the challenges of the current environment e.g. free air space vs. cluttered habitat^3^.

A dynamical adjustment of the sonar beam has been demonstrated earlier for vespertilionid and rhinolophid bats^21^,^22^. Here, the reported broadening of the sonar beam (decrease directionality) is task dependent and occurs during the final stage of prey capture. Jakobsen and Surlykke^21^ demonstrated that for mouth-emitting vespertilionid bats, the sonar-beam broadening is a by-product of the pronounced lowering of the spectral centroid of the calls during the final buzz II. While hunting fast-moving, aerial prey, broadening the sonar beam is advantageous, because the bats need to ensonify the largest possible near-field airspace shortly before capturing the prey in order not to miss it. Rhinolophid bats also broaden the beam (of the call FM components) when homing in on prey. However, these nostril-emitting bats keep the spectral content of their signals constant but seem to modify the shape of the nose leaf to achieve the sonar beam broadening^22^,^26^. The mechanisms behind this strategy are yet not fully understood. Another possibility to adjust beam directionality for mouth emitting bats is the modification of the mouth gape^25^. Increasing the mouth gape, while leaving the spectral centroid constant, sharpens the beam.

On top of the large range in sonar beam measures, our data show that three of the six bats studied systematically sharpen their sonar beam, both in beam height and width, when focusing onto the approaching object. At first sight, this beam sharpening appears counter intuitive because the angular aperture taken up by the object approaching the bats increases with decreasing distance. Indeed, many bats have been observed to widen their beam when approaching a prey object in flight, which is in contrast to our findings. Surlykke *et al*.^28^ have shown that in the phyllostomid bat *Trachops cirrhosus*, a bat with a very similar echolocation call design and nose leaf as *P. discolor*, the sonar beam is generally quite sharp (directionality index around 17 dB) and, like for *P. discolor*, echolocation calls are relatively faint (around 88 dB SPL when perching). Thus, the hunting mode (perching vs. in flight) appears related to the echolocation call directionality and its dynamics. Perching animals appear to emit the more directional and fainter calls. In this situation, a sharper beam leads to a more effective suppression of echoes from off-axis (clutter) reflectors, and an improved object localization: both when pointing the main axis of the sonar beam onto the object^25^, or when bats chose to alternating point off-axis to facilitate the analysis of aim-dependent variations in echo level^39^, bats benefit from a sharper sonar beam.

All bats in the current experiments reduced the echolocation call level and –duration with decreasing object distance. These reductions lead to a reduction in sonar search volume, i.e., the size of the space from which an echo of the call may be detected. Those three bats that combined this level- and duration reduction with beam sharpening reduce the sonar search volume even more, because at the same call level (that is always taken from the loudest microphone signal on the array) the sharper call covers less space. Thus it appears that sonar beam sharpening is a further, facultative means of reducing search volume, likely to be employed by stationary animals when the object position is close and unambiguous. This hypothesis is corroborated by experiments with a stationary and echolocating false killer whale, that showed that this whale also sharpened its sonar beam when inspecting properties of an object presented close by^38^. Hence, it could be that the results observed in these studies, beam sharpening with decreasing distance of interest, are partly driven by the experimental design and the behavioural context.

Brinklov *et al*.^15^ did not find sonar beam dynamics for a phyllostomid bat (*Carollia perspicilata*) although some of their observations suggest beam widening when flying towards a landing platform (see their Fig. 4 and corresponding legend). A detailed analysis on the individual level may have revealed individual based strategies. Thus we can only speculate that for the remaining three bats, that did not adjust their sonar beam to object distance, like the bats in the Brinklov *et al*.^15^ study, the experimental task was not challenging enough or they used a different strategy. An object discrimination task at fixed distances could probably force the bats to focus their sonar beam onto the object. Therefore, the results indicate that the beam sharpening is under voluntary control of each animal and indicate that individuals may follow different preferred approaches to solve an echolocation task.

The actual active sensing in echolocation takes place in the auditory system. Thus any modification of the emitted signal is purely meant to facilitate auditory processing of the corresponding echoes. Narrowing the sonar beam has the advantage of limiting undesired echoes of clutter surrounding the object of interest. Further it improves object detection as a higher proportion of the emitted energy is focused onto the object. Overall directionality of an echolocation system is the product of the emission and reception directionalities. Thus bats may also adjust the directionality of their ears to object distance. Unfortunately, manipulating the reception (ear) directionality cannot be observed with a microphone array. Instead, complex 3D reconstruction of outer ears, and directionality computations from there must be performed^30^. This remains a challenge for future studies on bat echolocation.

We observed a small increase of the call spectral centroid with decreasing object distance for all six bats. However, this change cannot account for the sonar beam sharpening because only three out of the six bats sharpened their beam significantly with decreasing object distance. Also beam sharpening cannot occur by simply lowering the call level, as we did not find a correlation between any beam parameter and call level (supplementary information, Fig. SI2). We recently demonstrated^40^ that *P. discolor* can control different echolocation call parameters (such as call level, duration and frequency content) independently of each other. Manipulating sonar beam shape by means of changes in the spectral content of the echolocation calls comes at another cost: the mammalian peripheral auditory system operates with constant Q filters, meaning that the width of auditory filters is a constant percentage of the filter centre frequency and the narrower a filter, the worse its temporal resolution^41^. Thus, lowering the call spectral centroid decreases the temporal resolution of auditory echo processing. This is for instance the trade off in the case of prey capturing vespertilionid bats during the final buzz II. Maintaining a high spectral centroid, where spectral resolution is not so good but temporal resolution is good, appears advantageous for the auditory-neural analysis of echo delay. To keep a good temporal resolution but simultaneously sharpening the sonar beam onto the object of interest, bats have to adjust their sonar beam in a different manner than by frequency. Some of our animals sharpened their sonar beam by more than 10° when the object approached from a distance of about 60 to 10 cm (Fig. 3). Simultaneously, these bats increased the spectral centroid of their echolocation calls from ca. 65 to 67 kHz (Fig. 4). To test whether this frequency increase is sufficient to explain the sonar beam focusing, we used the formula of Jakobsen and Surlykke^21^ to calculate sonar beam directionality. Although the formula is based on mouth-emitting bats (single sound source) it allows us to roughly approximate sonar beam directionality for nose-emitting bats (two sound sources) and thus demonstrate the mechanism behind the theory on frequency dependent sonar beam dynamics. Based on a constant 4 mm radius^30^ of the sound source (nostrils surrounded by nose leaf) and a spectral centroid of 66.8 kHz, we confirmed the measured beam shape of 40°. Increasing the centroid from 65 to 67 kHz, as observed in our data, results in a decrease of the beam shape by only 1°, which is much less than experimentally observed. Indeed the spectral centroid would have to be increased from 65 to about 85 kHz to focus the beam by 10°, as observed in the sonar beam-pattern data (Fig. 4). Therefore, unlike it has been described for vespertilionid bats, the focusing of the sonar beam in *P. discolor* is not mediated by frequency changes.

Preliminary data from our lab indicate that both lateral parts of the nose leaf, flanking the nostrils, as well as the tip of the nose leaf are movable relative to the head and these movements may underlie frequency-independent beam forming in *P. discolor*. Thus, we hypothesise that sonar-beam focusing could be mediated by a change in the shape of the emitter, in the case of *P. discolor* the nose leaf. This mechanism would be functionally equivalent to the adaptive change in the shape of the nose leaf in rhinolophid bats (but with the opposite result^22^) or the shape of the melon of toothed whales (both with equal sign^38^ or opposite sign^19^). This hypothesis is supported by earlier work^29^,^30^, which showed that in nose-emitting bats, the nose leaf defines the height of the sonar beam while the spacing of the nostrils defines its width.

We conclude that phyllostomid bats (*P. discolor*) have a highly dynamical biosonar. We find that these bats decrease the call level, the call duration, and the inter-call interval of their sonar calls with decreasing object distance. The sonar beam height and width are highly variable for all animals and independent of changes in call spectral content or call level. Our data highlight that the sonar beam plays an important role in bat biosonar, it can be voluntarily adjusted over a large range, and the current experimental task motivated some of our bats to sharpen their sonar beam with decreasing object distance. Thus it appears that sonar-beam sharpening is a further, facultative means of reducing search volume, likely to be employed by stationary animals when the object position is close and unambiguous. We suggest that beam sharpening is mediated by muscular control of the acoustic aperture of the bats’ nose leaf.

## Additional Information

**How to cite this article**: Linnenschmidt, M. and Wiegrebe, L. Sonar beam dynamics in leaf-nosed bats. *Sci. Rep*. **6**, 29222; doi: 10.1038/srep29222 (2016).

## Supplementary Material

Supplementary Information
