# Supplementary material for: Sonar beam dynamics in leaf-nosed bats
Source: Sci Rep. 2016 Jul 7;6:29222. doi: 10.1038/srep29222 (PMC4935842; doi:10.1038/srep29222)
Supplement: Supplementary Information [file srep29222-s1.pdf]

Supplementary information

Sonar beam dynamics in nose-leafed bats

Meike Linnenschmidt and Lutz Wiegrebe

Ludwig-Maximilians-University Munich, Division of Neurobiology, Dept. Biology II, Großhaderner Str. 2, 82152 Planegg-Martinsried, Germany

Correspondence and requests for materials should be addressed to M.L. (email: meike.linnenschmidt@gmail.com)

Supplementary methods

Array microphone calibration. An ultrasonic loudspeaker loudspeaker (D2004/602000, Scan Speak, Videbaek, Denmark) playing random noise (35 kHz high pass filter) for 4 s was placed 10 cm in front of each microphone and a reference microphone (1/4 inch Brüel & Kjær, Næum, Denmark). Cross-correlations between each microphone recording and the reference microphone generated frequency dependent compensation coefficients for each microphone for individual calibration.

*Set up calibration***.** An experiment with an ultrasonic loudspeaker (D2004/602000, Scan Speak, Videbæk, Denmark) at the bats' position continuously transmitting random noise (35 kHz high pass filter) was conducted to calibrate the setup. Acoustic impulse responses between the speaker and each of the 45 microphones were measured every 50 ms as a function of the object position. Impulse responses were analyzed in exactly the same way as the bats' echolocation calls (see materials and methods).

Supplementary figures and legends

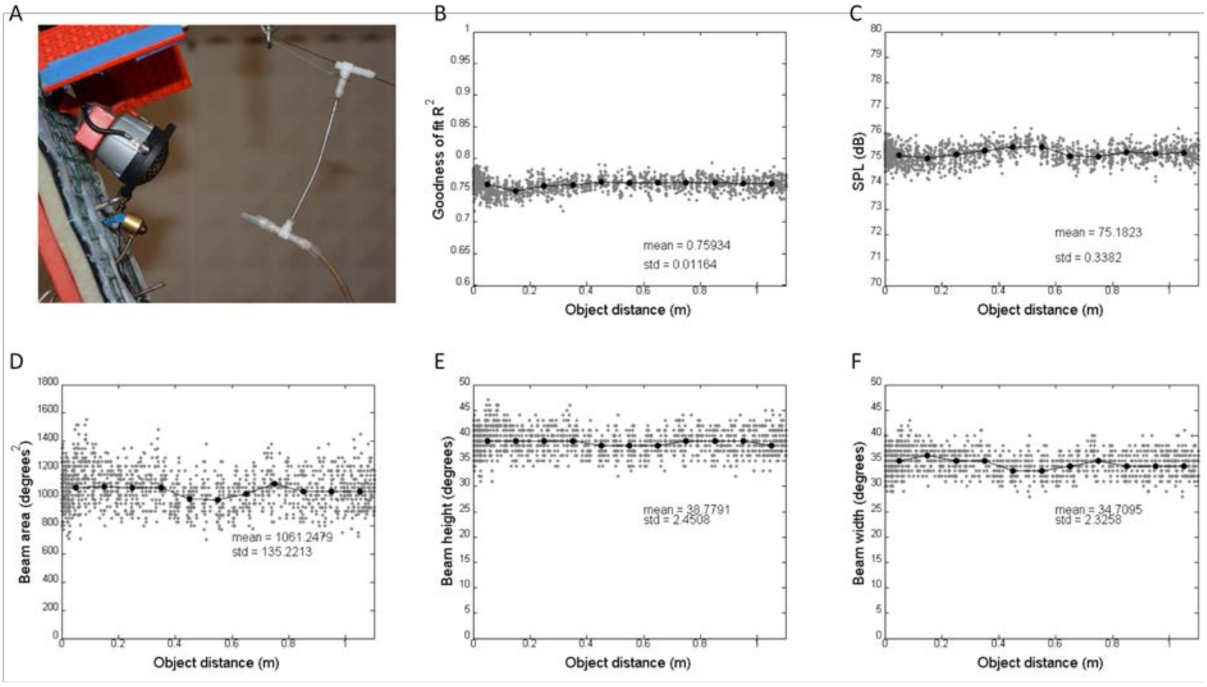

Figure SI1. Set up calibration. (A) Photo of loudspeaker in bats position and object (T-connector) in front of loudspeaker. (B) Goodness of fit of 2D Gaussian function to data, (C) sound pressure level (SPL) of loudspeaker, (D) sonar beam area, (E) sonar beam height, and (F) sonar beam width as a function of object distance. Mean value and standard deviation (std) are given in each subplot.

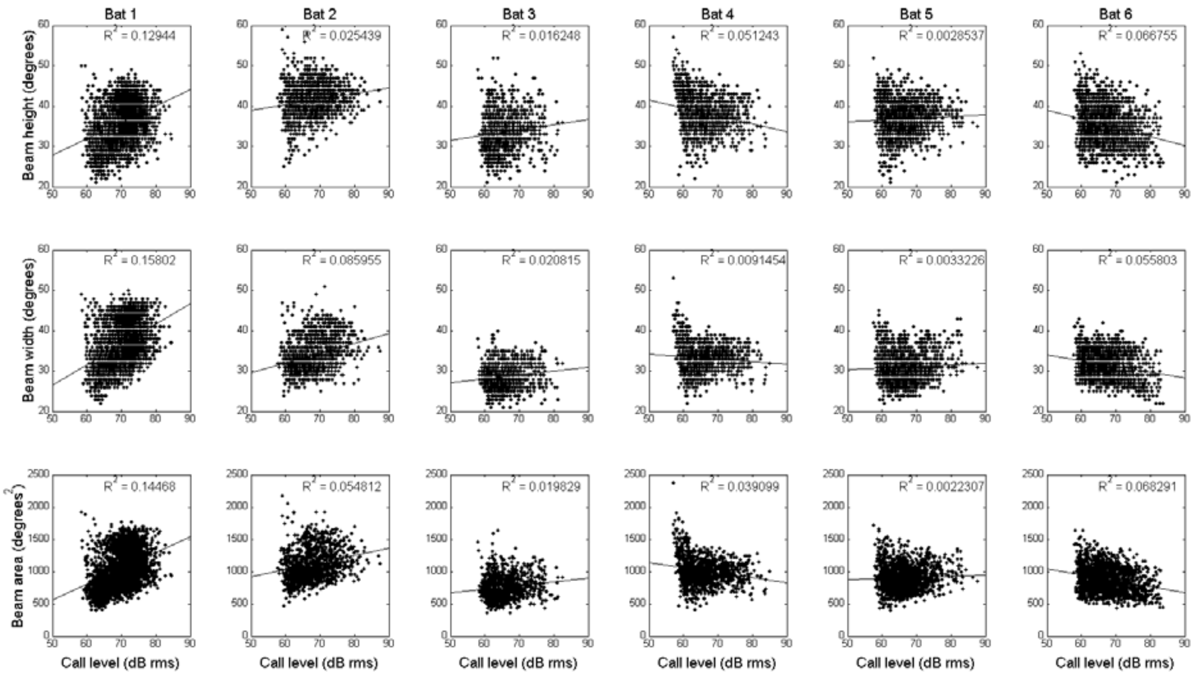

Figure SI2. Spatial sonar beam analysis vs. echolocation call level. The sonar beam height (upper row), beam width (middle row), and beam area (lower row) as a function of call level. Data is given for each bat separately. Note the very small R^2 values.
